# Tailored Care for Dementia Patients with Cerebrovascular Pathology

**DOI:** 10.3390/geriatrics10030081

**Published:** 2025-06-13

**Authors:** Sara A. J. van de Schraaf, Majon Muller

**Affiliations:** 1 Geriatrics Section, Department of Internal Medicine, Amsterdam UMC, Location Vrije Universiteit Amsterdam, De Boelelaan 1118, 1081HV Amsterdam, The Netherlands; s.vandeschraaf@amsterdamumc.nl; 2 Amsterdam Cardiovascular Sciences, 1081HV Amsterdam, The Netherlands

With the growing incidence of dementia among patients, optimal care for patients and caregivers is becoming crucial. Patients with cognitive disorders often become dependent on care in daily activities, which is largely provided by informal caregivers who often perceive caregiving as burdensome [1]. Due to patients wanting to live at home for as long as possible, sufficient formal care is crucial.

In Western countries, vascular cognitive impairment (VCI) is the second most common cause of clinically diagnosed dementia. In addition, 30–70% of patients with diagnosed Alzheimer’s disease have significant cerebrovascular pathology [2]. Patients with cerebrovascular pathology or VCI have symptoms that are distinctive from typical Alzheimer’s disease. In these patients, cerebrovascular pathology can cause impairment in cognitive domains such as processing speed, but can also cause other symptoms such as motor and mood symptoms [3].

Results from the Amsterdam Ageing Cohort confirm this [3]. Of *N* = 315 patients with a dementia diagnosis, 38% had no/mild cerebrovascular pathology (Fazekas 0/1), 36% had moderate cerebrovascular pathology (Fazekas 2), and 26% had severe cerebrovascular pathology (Fazekas 3). Within this cohort, the presence of typical Alzheimer’s disease symptoms, such as memory and executive impairment, were not dependent on the severity of the cerebrovascular pathology (Table 1). However, dementia patients with a severe cerebrovascular pathology had a significantly reduced processing speed, more symptoms of apathy and a reduced gait speed compared to dementia patients with no or mild cerebrovascular pathology (Table 1).

Slower thinking (reduced processing speed), mood (more apathy symptoms), and walking (reduced gait speed) are interrelated and often mistaken as normative features of human aging [3]. However, we believe that extreme slowing is pathological, as it is associated with cerebrovascular pathology and adverse outcomes such as mortality, cardiovascular events, or functional decline [3,4,5]. In addition, earlier studies have suggested that these slowing symptoms, associated with cerebrovascular disease, are related to a higher burden on the caregiver [1].

Available dementia care structures mainly focus on patients with typical Alzheimer’s disease symptoms and not on the slowing symptoms. We emphasize the importance of identifying the presence of slowing symptoms in patients with cerebrovascular pathology and tailoring the care needs of these patients and their caregivers. The needs and wishes of these patients and their informal caregivers regarding care could be improved by providing tailored information, promoting awareness of neuropsychiatric symptoms, particularly apathy, and by healthcare professionals providing more guidance during decision-making [6]. Further, the current care structure for dementia patients with a co-morbid cerebrovascular pathology is fragmented, unstructured, largely reactive, and provided by various professionals [7].

Therefore, a coordinated, proactive, multidisciplinary approach tailored to the specific wishes of the patient and caregiver is necessary to fulfil the care needs of dementia patients with a cerebrovascular pathology. Tailored care could ultimately improve the quality of life for these patients and their caregivers, as well as save costs.

Connecting all relevant professionals is a major challenge. Innovative solutions, such as digital multidisciplinary care platforms, have the potential to aid the current care structure in becoming more comprehensive and integrated.

## Figures and Tables

**Table 1:** Relation between presence/extent of cerebrovascular pathology (white matter hyperintensities) and clinical symptoms in *N* = 315 patients with a dementia diagnosis.

	White Matter Hyperintensities		
	No/Mild (Fazekas 0/1) *N* = 118	Moderate (Fazekas 2) *N* = 113	Severe (Fazekas 3) *N* = 84
Memory (z-score)	Ref	0.04 (−0.14; 0.22)	0.12 (−0.08; 0.31)
Executive function (z-score)	Ref	0.17 (−0.15; 0.49)	0.18 (−0.17; 0.52)
Processing speed (z-score)	Ref	−0.38 (−0.68; −0.08) *	−0.45 (−0.77; −0.14) **
Apathy (symptoms) ^¥^	Ref	0.21 (−0.05; 0.47)	0.43 (0.15; 0.72) **
Gait speed (m/s)	Ref	−0.04 (−0.29; 0.22)	−0.46 (−0.75; −0.18) **

Adjusted for age, sex, and education. * *p* < 0.05; ** *p* < −0.01. ^¥^ apathy was measured through the GDS subscale (score 0–3).
